# If I’m Worrying About Getting Well, How Can I Get Well? Decision-Making among African Americans with Advanced CKD

**DOI:** 10.1093/geroni/igaa057.3368

**Published:** 2020-12-16

**Authors:** Tyrone Hamler, Megan Schmidt-Sane, Anne Huml, Mirela Dobre, Donna Taylor

**Affiliations:** 1 Case Western Reserve University, Cleveland, Ohio, United States; 2 University of Sussex, Brighton, England, United Kingdom; 3 Cleveland Clinic, Cleveland, Ohio, United States; 4 University Hospital, Cleveland, Ohio, United States

## Abstract

Objectives: Chronic kidney disease (CKD) is an emerging major public health concern in the United States. Shared decision-making (SDM) has gained attention as an important area of inquiry in chronic kidney disease research. Few studies focus on shared decision-making or preferences of older African Americans during the advanced stages of CKD before the initiation of dialysis. The objective of this study was to understand decision-making preferences and shared decision-making among older African Americans with advanced chronic kidney disease who have yet to start dialysis. Methods: Data were collected from an outpatient clinic sample of older African Americans ≥ 55 years old (N = 10) diagnosed with advanced CKD. Participants were administered a survey with open-ended questions related to shared decision-making, CKD healthcare and general healthcare preferences (both open-ended and closed-ended questions), and participant characteristics. A thematic analysis framework was applied to identify themes and patterns in the data. Results: Several themes emerged in regard to shared decision-making and patient preferences including: complexity of CKD management, uncertainty of prognosis, barriers and facilitators to CKD self-management and SDM, diagnosis and dialysis information, elements of SDM, and the structural and social context of SDM related to racial inequities. Discussion: Participants identified a nuanced understanding of the concerns related to managing CKD. The complex and ever-changing nature of CKD was emphasized as participants discussed how they perceived their care needs. This study provides implications for social work practice, healthcare policy and interprofessional collaboration in the care of older African Americans.

